# Fast walking with swing extension assistance in a nominally passive semi-powered prosthetic knee

**DOI:** 10.3389/frobt.2026.1747721

**Published:** 2026-03-19

**Authors:** David M. Marsh, Aaron Haake, Michael Goldfarb

**Affiliations:** Center for Rehabilitation Engineering and Assistive Technology, Department of Mechanical Engineering, Vanderbilt University, Nashville, TN, United States

**Keywords:** fast walking, knee prosthesis, powered knee prosthesis, speed walking, transfemoral amputee

## Abstract

Typical microprocessor-controlled knee prostheses (MPKs) are energetically passive devices. Like MPKs, the biological knee joint is also largely passive during the swing phase of walking. However, unlike users of MPKs, at fast walking speeds otherwise healthy individuals without amputation supplement knee extension during swing phase with power at the knee joint, presumably to increase the speed of knee extension. This paper examines the effect of employing powered swing extension assistance in an otherwise passive MPK at fast walking speeds. Three participants with transfemoral amputation were recruited to walk at speeds ranging from their self-selected walking speed to their maximum safe walking speed with and without powered swing extension assistance on a treadmill. Swing extension assistance was shown to result in a notable increase in the knee angular velocity during swing extension. Rather than translating to a reduction in swing extension time during overground walking, the additional time afforded by faster swing extension resulted in an increase in the time between full knee extension and the onset of loading, named the latency period, indicating users appear to prefer more assurance that their knee has reached full extension prior to heel strike while walking at their maximum safe walking speed. All three participants commented positively about the extension assistance during certain walking trials, but all three also found the elevated terminal impact during the end of swing bad, particularly below their maximum safe walking speeds.

## 1 Introduction

The ability to walk at a wide range of speeds is a key aspect of mobility. For people with non-vascular-related transfemoral amputation, the inability to walk fast is a frequently self-reported issue limiting mobility (Hagberg and Brånemark, 2001). Further, people with unilateral transfemoral amputation walk slower than people without amputation and people with transtibial amputation. The causes of this deficit are multifaceted, and as Genin et al. (2008) identifies, can be different for different people, and may also be device-dependent. For some of the participants in Genin et al. (2008), maximum sustainable walking speed was limited by fatigue in specific muscle groups, while for others, it was limited by the speed at which their knee could swing forward. This maximum walking speed limitation, particularly of mechanical prosthetic knees, is further discussed by Frigo and Tesio (Frigo and Tesio, 1986).

People with transfemoral amputation exhibit significantly increased swing time on the prosthesis side relative to the unaffected side (Boonstra et al., 1994; Highsmith et al., 2010), which is potentially a limiting factor in walking speed (i.e., waiting on the prosthesis to complete swing phase). Non-amputee ambulators typically both linearly increase their step length and linearly decrease their step time as a function of walking speed in order to walk above their self-selected speed (Latt et al., 2007). Doing so requires them to increase their hip range of motion while decreasing their swing time, and as identified in Marsh et al. (2024b), this swing time must be decreased while the knee has either equivalent or larger peak knee flexion angles, thus requiring much higher knee angular velocities.

One difference between healthy biological knees and conventional prostheses is that the healthy knee is capable of exerting power in swing phase during fast walking, while conventional prosthetic knees (e.g., those characterized in Boonstra et al., 1994; Highsmith et al., 2010) are not. Although swing phase knee motion in healthy walking has been characterized as primarily passive (e.g. Mochon and McMahon, 1980a; 1980b), we hypothesized that at high enough walking speeds, non-amputees may actively extend their knees during swing extension to further reduce swing time. In order to investigate, we analyzed the open-source walking data from healthy adult walkers made available by Camargo et al. (2021). The results of this analysis are presented in Section 3.1 Knee Extension Power as a Function of Walking Speed in Healthy Human Walking.

There are a number of prior works employing powered knee extension during walking with prosthetic knees (e.g. Sup et al., 2008; Tucker et al., 2015; Ingraham et al., 2016; Hood and Lenzi, 2018; Tran et al., 2022; Best et al., 2023; Gehlhar et al., 2023). There have also been several works focused on layering powered swing phase behavior on primarily-passive swing phase behavior (e.g. Park et al., 2016; Baimyshev et al., 2018; Lee et al., 2020; Bartlett et al., 2022; Culver et al., 2022; Fu et al., 2023; Lee and Goldfarb, 2023; Marsh et al., 2024b). However, none of these prior works explicitly examines the effect of adding power during swing phase knee extension on fast walking biomechanics, relative to fast walking with strictly passive swing phase knee extension (i.e., the swing extension of conventional knee prostheses). Specifically, based on the data shown in Section 3.1, we conjecture that adding power to a nominally passive swing extension movement will decrease swing extension time during fast walking, and as a result may enable safer and/or preferred fast walking for people with transfemoral amputation.

### 1.1 The latency period: a useful metric for measuring swing completion

In their work examining walking in people with transfemoral amputation, Mâaref et al. (2010) characterize the time period between full knee extension during swing phase and leg loading as the latency period. Their study examined 29 participants with transfemoral amputation and 15 healthy participants, and it found that healthy controls walked with essentially no latency period (15 ms ± 9 ms) at their self-selected pace, while those with unilateral transfemoral amputation walked with substantial latency periods (110 ms ± 60 ms) at their self-selected pace.

They conclude by asserting that latency period may be an inverse indication of walking confidence, where a short latency period implies a high degree of walking confidence and a long latency period implies low confidence. In other words, a longer latency period provides increased assurance that the knee is fully extended (and thus ready to accept the bodyweight without buckling) prior to heel strike. While this provides an important exploration of swing extension in participants with transfemoral amputation, the study only investigated each participant at their own self-selected cadence. Therefore, the study does not inform how the latency period might change as a function of walking speed. Specifically, if walking speed with a transfemoral prosthesis is limited by swing extension time, one might conjecture that the latency period would necessarily decrease with increased walking speed. No other work exists that directly informs this conjecture, so we investigated it using data from an open source dataset (Hood et al., 2020) in Section 3.2 prior to completing our experimental procedure. The result of this analysis implies prosthesis users may have a minimum latency period (a minimum level of assurance) that each is willing to accept. If swing time is insufficient to provide this latency period, users may not be comfortable walking at this speed or faster.

### 1.2 Hypothesis

As presented in Section 3.1, walking data from Camargo et al. (2021) indicates that healthy, non-amputee individuals employ powered swing extension at fast walking speeds (i.e., above around 1.2 m/s), presumably to reduce swing time to accommodate the increased cadence associated with fast walking speeds. Individuals with transfemoral amputation walking on energetically passive prostheses cannot exert powered assistance and therefore may be limited in fast walking by the limited velocity of knee extension. It is hypothesized that the introduction of powered swing extension assistance will enable prototype users walking substantially above their self-selected walking speed to increase their cadence or increase the latency period at the end of swing extension. This work is designed to illuminate both the biomechanical effects of adding swing extension power as well as the user acceptance of one proposed method of doing so.

## 2 Materials and methods

### 2.1 Experiment overview

**FIGURE 1:**
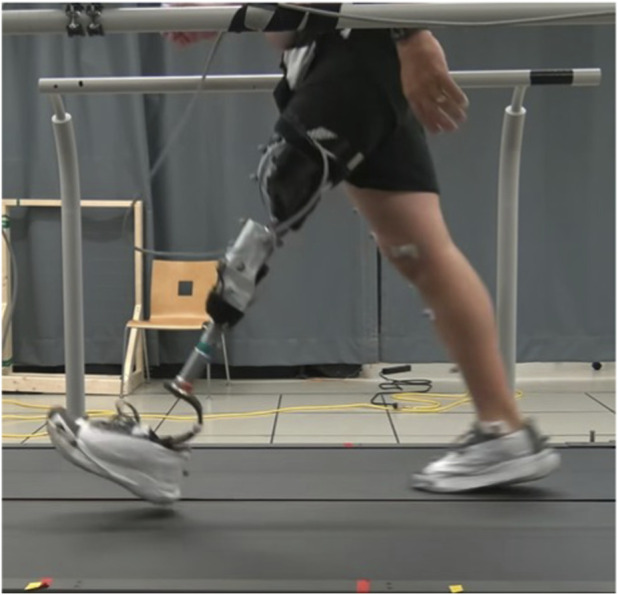
Participant 3 walking fast on the split belt Bertec treadmill with Vicon reflective markers. This image also depicts the knee during the latency period. Notice that the knee is fully extended while the prosthesis side foot has not yet struck the ground.

The authors conducted a set of experiments to examine the effects of power-assisted swing extension on fast walking. In particular, the authors sought to examine the effect of power-assisted swing extension on swing extension velocity, stride and swing time, and latency period during fast walking at three different speeds: self-selected speed, the maximum safe walking speed, and halfway between. The experiments are designed to compare walking with conventional passive knee extension (i.e., MPK-like knee extension) with power-assisted extension, both utilizing the same prototype knee prosthesis. Figure 1 provides a representative participant walking at their maximum safe walking speed while using the prototype knee prosthesis. Inclusion criteria required that participants be capable of walking at a treadmill speed 50% above their self-selected speed (e.g., for a participant with a 0.9 m/s self-selected speed, the participant was required to be capable of walking at least 1.35 m/s). Demographic information regarding the three participants is given in Table 1. The experimental protocol was approved by Vanderbilt IRB #110667.

**TABLE 1:** Relevant participant demographic information.

Participant ID	P1	P2	P3
Gender	Male	Male	Male
Age	31	37	57
Years since amputation	6	6	14
Height (meters)	1.73	1.85	1.83
Weight (kg)	104	108	91
Daily use knee	Ottobock C-Leg	Proteor Quatro	Ottobock X3
Daily use ankle	Fillauer AllPro	Fillauer AllPro XTS	Fillauer AllPro
Self-selected treadmill walking speed (m/s)	0.8	0.9	0.8
Max safe treadmill walking speed (m/s)	1.5	1.4	1.4

Experiments were conducted on three people with transfemoral amputation walking on a Bertec instrumented treadmill with six axis force plates (1000 Hz) with a VICON motion capture system (200 Hz). A custom lower body plus torso marker set was utilized on each participant (4 marker foot segment, 2 marker ankle joint, 4 marker shank segment, 2 marker knee joint, and 4 marker thigh segment per leg, in addition to a 4 marker hip joint and 6 marker back segment). Raw marker data were imported from VICON Nexus into Visual3D for kinematic and dynamic analysis.

**FIGURE 2:**
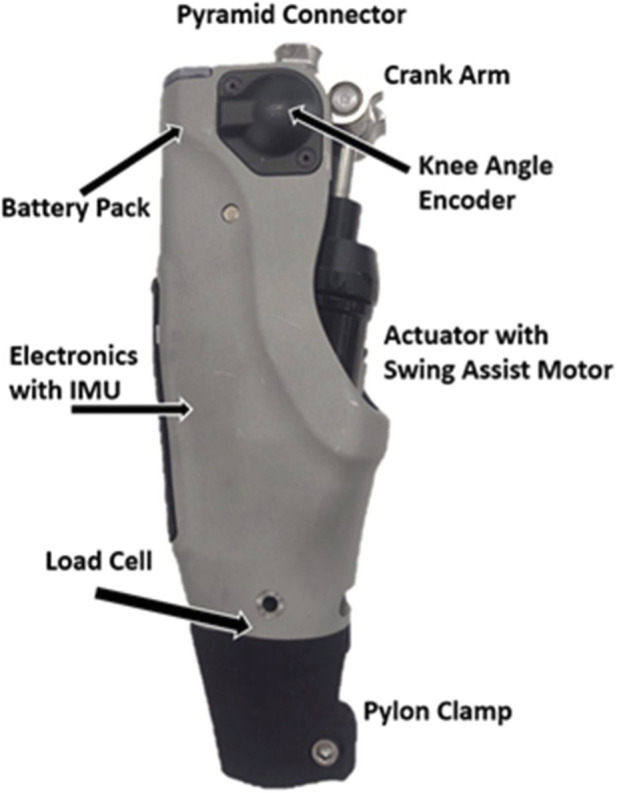
SCSA V2 prosthetic knee prototype, with major components identified. This image is taken from Marsh et al. (2024b), licensed under CC BY 4.0.

The Stance Control Swing Assist (SCSA) V2, the knee prosthesis prototype used in the experiments and shown in Figure 2, was previously described in Marsh et al. (2024b), which also confirmed that its passive walking characteristics are representative of conventional passive MPKs (see Figures 9-11 of Marsh et al. (2024b)). In the baseline unassisted case (conventional MPK knee extension) the knee extends under a combination of gravity and inertial coupling with the thigh, where the knee acts like a lightly damped hinge with an extension bias, which is typical of MPKs. The hydraulic damping was configured to be minimal during most of swing extension, with a rapid increase in damping over the final 20° of knee extension to prevent terminal impact.

### 2.2 Swing extension controller

In the swing-assisted knee extension case, the SCSA V2’s swing-assist motor was used to provide supplemental power to assist knee extension. The control law used to add supplemental power was a modified version of the “negative damping” controller used in the authors’ prior work (Marsh et al., 2024b), although applied to the swing extension gait phase, rather than the swing flexion gait phase (for the state machine used in this work, see Marsh et al. (2024b)). (Equation 1) provides the control law as implemented in this work,

$$F_{ext}=\beta_{ext}\sqrt{\left|\dot{x}\right|}$$ (1)

where $F_{ext}$ is the commanded extension force, $\beta_{ext}$ is the extension assistance gain, and $\dot{x}$ is the measured speed of the SCSA V2’s piston (in m/s). $F_{ext}$ is related to the knee torque during swing extension $T_{ext}$ by the transmission ratio of the SCSA V2, which is given in Marsh et al. (2024a).

This control law is only used during swing extension, and it does not interfere with the user’s interaction with the prosthesis during stance phase or swing flexion, effectively modifying only the knee extension gait phase, without otherwise changing the behavior of the knee. Because the healthy non-amputee knee extension torque (see Section 3.1) is positive during early swing extension (where extension velocity is lower), the control law employs a square root function of piston velocity rather than a linear or quadratic one to more heavily weight forcing at lower piston velocities. To smoothly transition between generative knee torque (i.e., extension assistance) and resistive knee torque that slows the knee down to lessen terminal impact, the extension assistance gain $\beta_{ext}$ is linearly reduced to 0 between a knee angle of 35° and 20°. Around 20°, the underlying passive behavior of the knee provides increased hydraulic damping for the remaining portion of swing extension. Experiments employed three values of extension assistance gain, one zero (the control case), one lower ( $\beta_{ext}=225$ ) and one higher ( $\beta_{ext}=450$ ). As such, through the course of these experiments, each participant walked using the knee prosthesis prototype at three fast walking speeds and with three different knee behaviors (no assist, low assist, and high assist), for a total of nine experiments each.

The authors hypothesized that the swing extension assistance would potentially increase cadence and/or the latency period in addition to the knee extension velocity. Because these experiments were completed on a treadmill at fixed walking speed, the step and swing times are related to the step length, so only two of these three parameters are independent. Step and swing times are the most easily-measurable and directly comparable gait parameters to the latency period, so the authors elected to report these metrics, rather than the step length.

### 2.3 Experimental procedure

For each participant, experiments began by first determining the self-selected and then the maximum safe walking speeds using daily-use prostheses while walking on the treadmill. The self-selected treadmill speed was found by starting the treadmill at 0.5 m/s and incrementing by 0.1 m/s. After each participant indicated they were at their self-selected speed, the treadmill speed was again increased by 0.1 m/s to ensure their preference was not higher. The maximum safe walking speed is defined in these experiments as “the maximum speed at which a participant would choose to walk given extenuating circumstances but not emergencies.” Participants were given the instructions, “this speed should be the speed at which you may rarely walk if you were late to an important meeting or about to miss a plane flight.” The self-selected and maximum safe walking speeds found for each participant are provided in Table 1.

After determining the participant’s self-selected and maximum safe walking speeds, the participant was provided with a brief overview of the experiment, including the purpose (to test swing extension power) and the number of trials (three at each speed: self-selected, an intermediate speed, and the maximum safe walking speed). The participant was informed that the experiments would be blinded for extension assistance, and after each trial they would be prompted to provide feedback about any similarities or differences, particularly during swing, with the behavior of the knee during an acclimation trial. The participant was then fit with the experimental prosthesis prototype combined with the same socket and daily-use foot used in the participant’s daily-use prosthesis. The participant was allowed to acclimate to the prototype in parallel bars for as long as they desired, though none took longer than 10 minutes. During this time, alignment of the prototype knee and daily-use ankle assembly was also confirmed. At the end of the parallel bar acclimation, the swing extension assistance was briefly turned on to test its functionality. Participants were told that they may feel the knee change behavior during this time, and that the prototype was functioning as intended.

A lower body plus torso marker set was used for recording motion. The experimental prosthesis prototype has been previously shown to offer improved knee flexion during walking, particularly at low walking speeds, with swing flexion assistance (Marsh et al., 2024b). The authors chose to employ swing flexion assistance in addition to swing extension assistance in these experiments for two reasons. First, swing flexion assistance helps make peak knee angle during swing healthy-mimetic across walking speeds. Second, since peak knee angle is the initial condition for swing extension, improved uniformity in peak knee angle also provides improved consistency of the initial conditions of the swing extension controller presented here.

Therefore, in order to establish the swing flexion assistance control gains to provide consistent healthy levels of peak knee flexion, each participant next walked for approximately 90 seconds at their respective self-selected speed before progressing through an intermediate fast walking speed to their maximum safe walking speed. During this trial, the authors hand-tuned the swing flexion assistance gains, using a rolling four stride median peak knee angle monitor implemented on the prototype. For full details of this controller structure, please see Marsh et al. (2024b). Each participant’s resulting swing flexion assistance gains are provided in Table 2. Participants were not blinded to this process; they were told flexion assistance values may change and that extension assistance was turned off during this tuning trial.

**TABLE 2:** Flexion assistance gains used during all tested extension experiments. A positive number indicates the knee provides assistive flexion torque at that speed, while a negative number indicates the knee provides resistive torque.

Participant	P1	P2	P3
Self-select speed flexion gain	1000	700	700
Intermediate speed flexion gain	750	200	200
Max safe speed flexion gain	500	−300	−300

Following establishment of swing flexion assistance control gains, each participant completed walking trials at each speed with flexion assistance turned on, and with various levels of extension assistance to provide a familiarity with the behavior of the knee. Participants were blinded to the levels of extension assistance during this time. A final acclimation trial, labeled “A” in Table 3, was completed with no swing extension assistance at the intermediate speed. Participants were informed that this acclimation trial was completed with flexion assistance but without extension assistance, and to focus on how it felt, as it would be the baseline when evaluating the behavior of the prosthesis during the experimental trials. Participants were informed that the provided prototype extension behavior during the acclimation trial was similar to that of passive hydraulic MPKs.

**TABLE 3:** Table of trial experimental settings, where trial A is the final acclimation trial and trials 1-9 are the main experiment trials.

Trial	Walking speed	P1 Ext. Gain	P2 Ext. Gain	P3 Ext. Gain
A	Int	0	0	0
1	Self	0	225	0
2	Self	225	0	450
3	Self	450	450	225
4	Int	450	225	0
5	Int	0	450	225
6	Int	225	0	450
7	Max	0	0	225
8	Max	450	450	450
9	Max	225	225	0

Then, nine 30-s walking experiments were completed. Because walking speed is non-blindable (participants immediately know at what speed they are walking) and the experiments required the participants to walk at elevated, difficult speeds, the authors elected to ramp up the walking speed in ascending order (self-select, intermediate fast, and max safe). To limit systematic biases, each of the three different values of swing extension assistance ( $\beta_{ext}$ = 0, 225, or 450) were randomized and blinded to each participant, with Table 3 providing each participant’s walking trial order. A video accompanies this work, providing sagittal views of all three participants with no extension assistance and full extension assistance for both their self-selected speeds and maximum safe walking speeds.

### 2.4 Data processing and statistical methods

The data presented in this work were post-processed with a zero-phase Fast Fourier Transform-based low-pass filter with a 30 Hz cutoff frequency. Strides were parsed using the measurement from the prototype’s onboard load signal, and misstep strides were removed from these data. The following step inclusion criterion were used to identify full, correct steps: i) steps must be started more than one second after the treadmill reached steady state speed and ended before one second prior to treadmill speed ramp down, ii) steps must not cross over the treadmill belt split at any time, even partially, and iii) step loading must register at least 94% body weight by 100 ms after initial heel strike load, to ensure steps with ground scuffs during late swing are discarded.

Because of the low number of recruited participants, we decided to limit our statistical analyses to only differences within each participant at each walking speed. The data in this work were analyzed in a case-study format, and population inferences are not possible. Therefore, participant to participant comparisons were not made, nor were comparisons between walking speeds made, and all discussions of statistical significance relate to differences in the presented data between the full assistance, half assistance, and no assistance cases for each participant and walking speed individually. All presented data were checked for normalcy using the Kolmogorov-Smirnov goodness-of-fit hypothesis test for normalcy (with significance level 0.05), and we have failed to reject the null hypothesis that the presented data are normal. This normalcy test was only completed for each trial individually. For example, we have failed to reject the null hypothesis that Participant 1’s peak knee flexion angle at his self-selected walking pace with full extension assistance (Participant 1’s Trial 3) is normal. This is true of every trial, participant, walking speed, and variable tested. All data were visually inspected qualitatively; no clear outliers were found. No formal outlier identification or removal processes were completed (other than the misstep rejection) because any quantitative outliers in the data represent real strides that we believe should be considered valid data.

Basic independent t-tests with assumed unequal variances were completed for each presented result. Statistical significance was set to p < 0.05 with Bonferroni correction of 27 for all statistical tests (the magnitude and phase of the peak knee angle, peak knee angular velocity, latency period, and swing and step times), because there are three participants, three walking speeds, and three comparisons made per investigated variable.

Processed but otherwise unparsed and unfiltered time series Visual3D data are provided at the following Digital Object Identifier (DOI): 10.5281/zenodo.18675888.

### 2.5 Participant questions after each walking trial and the conclusion of experiments

After each walking trial, participants were asked the following two questions:

1. Do you believe the behavior of the knee during this trial was different from its behavior during the acclimation trial?
2. If so, do you like the change in behavior, relative to the acclimation trial?

Additionally, after the conclusion of the final walking trial, the participants were asked four questions about the entirety of the experiment. These four questions were:

1. Overall, what are your thoughts about these walking trials?
2. Were there any trials in which you preferred the prototype’s behavior more than its behavior during the acclimation trial? If so, for these trials, what did you like about the prototype’s behavior?
3. Were there any trials in which you preferred the prototype’s behavior during the acclimation trial over the prototype’s trial behavior? If so, for these trials, what did you not like about the prototype’s behavior?
4. Do you have any final thoughts?

## 3 Results

### 3.1 Knee extension power as a function of walking speed in healthy human walking

**FIGURE 3:**
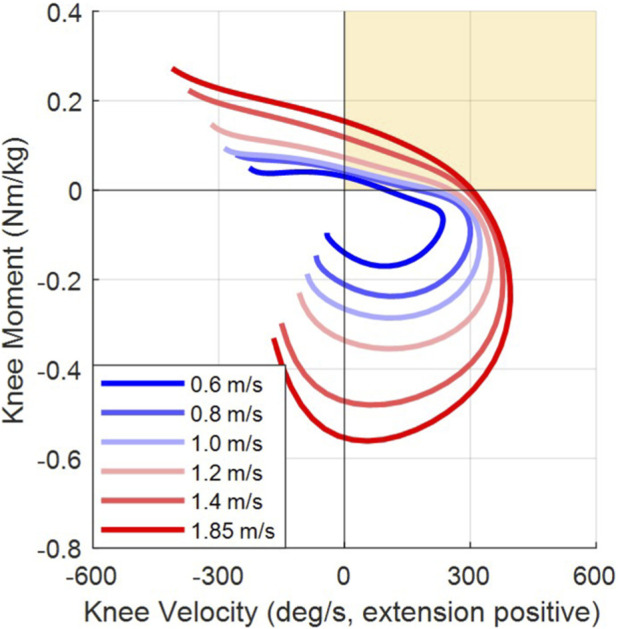
Power plane information of healthy walkers during swing at six walking speeds. Note that these curves progress clockwise from toe off in the second quadrant to heel strike in the third quadrant.

Figure 3 presents the average knee torque plotted versus knee angular velocity for these healthy, non-amputee participants walking at six different walking speeds. The first and third quadrants depict power generation by the knee, while the second and fourth quadrants depict power dissipation. Positive angular velocity denotes the extension phase, such that each segment starts in the second quadrant (at toe-off) and progresses clockwise from swing flexion to swing extension and ends with the slight knee flexion that (in healthy, non-amputee subjects) precedes heel strike (in the third quadrant).

Note that at walking speeds below 1.2 m/s, the Loci do not notably enter the first quadrant of the power plane, while above that speed they do. This is an indication in healthy non-amputee individuals that the knee exhibits active (or powered) early knee extension during fast walking. Late swing extension is always characterized by dissipation (in the fourth quadrant). Note also, that for healthy non-amputee subjects, terminal swing phase is characterized by powered knee flexion, since the knee actively begins to flex just ahead of heel strike, for purposes of ground speed matching.

These data support the observation that healthy non-amputee walkers employ positive knee power in early swing extension, presumably to decrease swing phase time during fast walking, while passive prosthesis users do not have access to this same powered behavior. Modern passive prostheses have extension bias springs which can slightly assist extension behavior, but such extension springs typically provide only around one to 2 N·m of extension torque, which is much lower than the 0.1 N m/kg observed in Figure 3 at 1.4 m/s (i.e., prosthetic knee extension springs are roughly an order of magnitude weaker than the torque observed in healthy participants during early swing extension of fast walking), (Toderita et al., 2025).

### 3.2 Latency period as a metric for swing completion

**FIGURE 4:**
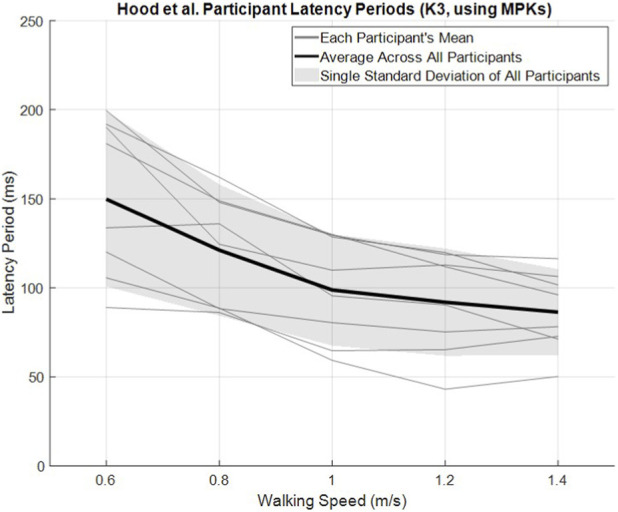
Hood et al. K3 latency periods of eight transfemoral amputee participants across five walking speeds.

In order to better inform the effect of walking speed on latency period for participants walking with a transfemoral prosthesis, the authors examined the open source dataset from Hood et al. (2020). Figure 4 provides the mean latency period as a function of walking speed for each of eight K3 (i.e., community ambulator) MPK users, walking at five speeds between 0.6 m/s and 1.4 m/s. The mean across the eight participants and plus/minus one standard deviation is provided by the dark line and the gray band, respectively. As seen in the figure, the mean latency period decreases as a function of walking speed, and then converges above approximately 1.1 m/s. Since the mean latency period appears to reach a minimum above approximately 1.1 m/s, these data may support the conjecture that the fastest confident walking speed may be limited by the minimum swing extension time, presumably resulting from a lack of powered knee extension during fast walking.

### 3.3 Knee angle during swing extension

**FIGURE 5:**
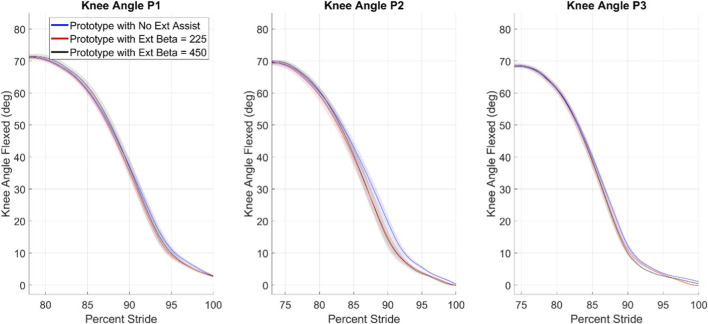
Swing extension knee angles for each participant at the fastest walking speed. The shaded regions surrounding each mean line is each mean line’s 95% confidence interval.

Figure 5 shows each participant’s mean knee angle during swing extension (as measured by the prototype’s onboard encoder) during their maximum safe walking speed trials for each of the three swing extension assist levels. As depicted in Figure 5, the initial conditions of swing extension are markedly similar within each participant and across the three participants. The magnitude and phase of each participant’s peak knee angles were evaluated with unpaired T tests with assumed unequal variances and a statistical significance of p < 0.05 with Bonferroni correction of 27 (the three extension assistance cases were compared with one another at each speed and for each participant). The full table of confidence intervals for these comparisons is provided by Table 4, with the four comparisons with statistical significance highlighted. None of those four differences were found in peak knee flexion angle magnitude nor phase for the maximum safe walking speed, meaning the initial conditions of the swing extension assistance controller were not statistically different in the maximum safe walking speed case. Differences in the other two walking speeds are small and inconsistent. Though the initial conditions are not statistically different, the mean extension trajectories with swing extension assistance (i.e., the red and black lines) lead the no extension assistance cases (i.e., the blue lines) for all three participants in Figure 5.

**TABLE 4:** Differences in peak knee angle across swing assist levels. The values in brackets are the confidence intervals (P < 0.05, Bonferroni correction of 27). The four statistically significant comparisons are shaded.

		P1			P2			P3		
	Ext. assist condition comparison	β = 0 β = 225	β = 225 β = 450	β = 0 β = 450	β = 0 β = 225	β = 225 β = 450	β = 0 β = 450	β = 0 β = 225	β = 225 β = 450	β = 0 β = 450
Parameter	Speed condition									
Peak flexion magnitude (degrees)	Self-selected	$\left[\begin{array}{c} -2.5\\ 0.5 \end{array}\right]$	$\left[\begin{array}{c} 0.2\\ 2.8 \end{array}\right]$	$\left[\begin{array}{c} -1.0\\ 2.1 \end{array}\right]$	$\left[\begin{array}{c} -2.5\\ 0.4 \end{array}\right]$	$\left[\begin{array}{c} -1.2\\ 2.5 \end{array}\right]$	$\left[\begin{array}{c} -2.3\\ 1.4 \end{array}\right]$	$\left[\begin{array}{c} -1.2\\ 1.3 \end{array}\right]$	$\left[\begin{array}{c} -1.2\\ 2.1 \end{array}\right]$	$\left[\begin{array}{c} -1.3\\ 2.3 \end{array}\right]$
	Intermediate	$\left[\begin{array}{c} -1.4\\ 1.3 \end{array}\right]$	$\left[\begin{array}{c} -0.3\\ 2.1 \end{array}\right]$	$\left[\begin{array}{c} -0.5\\ 2.1 \end{array}\right]$	$\left[\begin{array}{c} -0.6\\ 3.4 \end{array}\right]$	$\left[\begin{array}{c} -4.2\\ 0.2 \end{array}\right]$	$\left[\begin{array}{c} -2.2\\ 1.0 \end{array}\right]$	$\left[\begin{array}{c} -1.7\\ 0.3 \end{array}\right]$	$\left[\begin{array}{c} -1.2\\ 1.6 \end{array}\right]$	$\left[\begin{array}{c} -2.0\\ 0.9 \end{array}\right]$
	Max. safe	$\left[\begin{array}{c} -0.9\\ 1.4 \end{array}\right]$	$\left[\begin{array}{c} -2.0\\ 1.0 \end{array}\right]$	$\left[\begin{array}{c} -1.7\\ 1.2 \end{array}\right]$	$\left[\begin{array}{c} -2.0\\ 1.4 \end{array}\right]$	$\left[\begin{array}{c} -1.8\\ 1.4 \end{array}\right]$	$\left[\begin{array}{c} -2.1\\ 1.1 \end{array}\right]$	$\left[\begin{array}{c} -1.4\\ 1.1 \end{array}\right]$	$\left[\begin{array}{c} -0.8\\ 1.7 \end{array}\right]$	$\left[\begin{array}{c} -0.7\\ 1.4 \end{array}\right]$
Peak flexion phase (% stride)	Self-selected	$\left[\begin{array}{c} -1.7\\ 0.6 \end{array}\right]$	$\left[\begin{array}{c} -0.1\\ 2.2 \end{array}\right]$	$\left[\begin{array}{c} -0.7\\ 1.6 \end{array}\right]$	$\left[\begin{array}{c} -3.0\\ 1.2 \end{array}\right]$	$\left[\begin{array}{c} 0.1\\ 4.0 \end{array}\right]$	$\left[\begin{array}{c} -0.7\\ 3.1 \end{array}\right]$	$\left[\begin{array}{c} -1.6\\ 0.1 \end{array}\right]$	$\left[\begin{array}{c} 0.3\\ 2.1 \end{array}\right]$	$\left[\begin{array}{c} -0.6\\ 1.4 \end{array}\right]$
	Intermediate	$\left[\begin{array}{c} -1.1\\ 1.3 \end{array}\right]$	$\left[\begin{array}{c} -0.7\\ 1.2 \end{array}\right]$	$\left[\begin{array}{c} -0.7\\ 1.4 \end{array}\right]$	$\left[\begin{array}{c} -0.7\\ 2.3 \end{array}\right]$	$\left[\begin{array}{c} -3.1\\ 0.6 \end{array}\right]$	$\left[\begin{array}{c} -2.3\\ 1.4 \end{array}\right]$	$\left[\begin{array}{c} -0.9\\ 0.3 \end{array}\right]$	$\left[\begin{array}{c} 0.1\\ 1.2 \end{array}\right]$	$\left[\begin{array}{c} -0.2\\ 0.9 \end{array}\right]$
	Max. safe	$\left[\begin{array}{c} -0.8\\ 0.9 \end{array}\right]$	$\left[\begin{array}{c} -1.5\\ 0.2 \end{array}\right]$	$\left[\begin{array}{c} -1.5\\ 0.3 \end{array}\right]$	$\left[\begin{array}{c} -0.8\\ 1.3 \end{array}\right]$	$\left[\begin{array}{c} -1.7\\ 0.6 \end{array}\right]$	$\left[\begin{array}{c} -1.2\\ 0.7 \end{array}\right]$	$\left[\begin{array}{c} -0.8\\ 0.7 \end{array}\right]$	$\left[\begin{array}{c} -1.0\\ 0.6 \end{array}\right]$	$\left[\begin{array}{c} -0.8\\ 0.3 \end{array}\right]$

### 3.4 Amount of swing assistance

**FIGURE 6:**
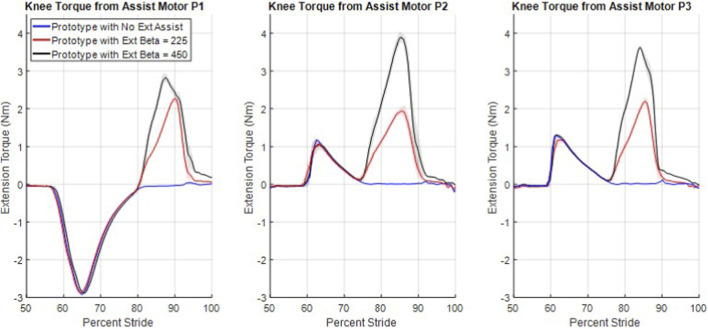
Knee flexion and extension swing assist torque. The first peaks, around 60 to 65 percent stride, occur during swing flexion and are the result of the swing flexion assistance, while the second peaks occur during swing extension, and are the result of the presented controller.

**FIGURE 7:**
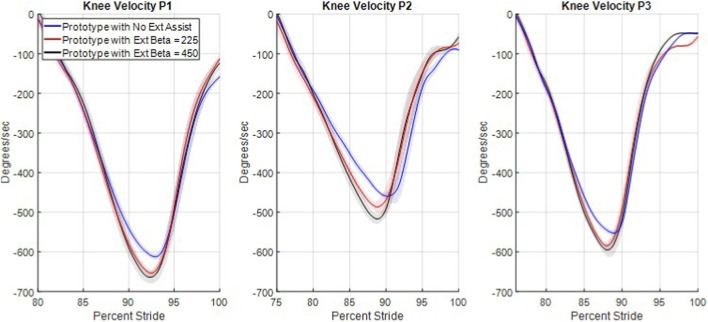
Swing extension velocity for each participant and extension assistance level while walking at their maximum safe walking speeds. The shaded regions around each mean line provide the 95% confidence intervals of the true location of the mean lines.

The swing flexion and swing extension assist torques at the maximum safe walking speed are shown in Figure 6. The first torque peak (which is negative for P1 and positive for P2 and P3) corresponds to swing flexion assistance, while the second torque peak (which is positive for all participants) corresponds to swing extension assistance at the three different assistance levels (no assistance in blue, medium in red, and maximum in black). All differences in the swing extension torque peak magnitudes are significant to P < 0.001. Note that, regarding flexion assistance, P1 has a positive flexion gain at the fastest walking speed, while P2 and P3 both have negative flexion gains (see Table 2). This is because P1 requires flexion assistance to achieve healthy levels of swing flexion, while P2 and P3 require flexion resistance. However, in extension all participants received extension assistance at fast walking, with the amount of assistance torque dependent on both the extension gain (i.e., no assistance, half assistance and full assistance) as well as the swing extension knee velocity, which is provided in Figure 7. Assistance ends around 90% stride, since terminal swing requires a deceleration to avoid terminal impact.

The amount of extension assistance provided to P1 (and to a lesser extent P3) at the highest level of assistance and at the maximum safe walking speed was back-EMF limited by the battery pack, as indicated by the asymmetrical and truncated peak of the black torque curve during extension in Figure 6 (around 85% stride).

### 3.5 Knee angular velocity

Figure 7 shows each participant’s mean knee angular velocity during swing extension for each of the three swing extension assist levels for the maximum safe walking speed trials. The angular velocity data show clear differences in knee angular velocity between the unassisted and fully assisted cases for all three participants at the maximum safe walking speed, which were similar to the other two walking speeds. For each of the three participants at each walking speed, the unassisted and full assisted peak knee extension velocities are statistically significant (P < 0.05, Bonferroni correction 27). The average increase in peak knee extension velocities for the three participants was 68 deg/s, 61 deg/s, and 60 deg/s at their self-selected walking speeds, intermediate walking speeds, and maximum safe walking speeds, respectively. Only four of the twenty-seven comparisons between the extension assistance cases were not found to be significantly different and all four were between the half assistance case and full assistance case: P1 self-selected speed, P2 self-selected speed, P3 intermediate speed, and P4 maximum safe speed. In all 18 comparisons between no swing extension assistance and either other level of swing extension assistance, statistically significant differences are seen.

### 3.6 Latency period

**FIGURE 8:**
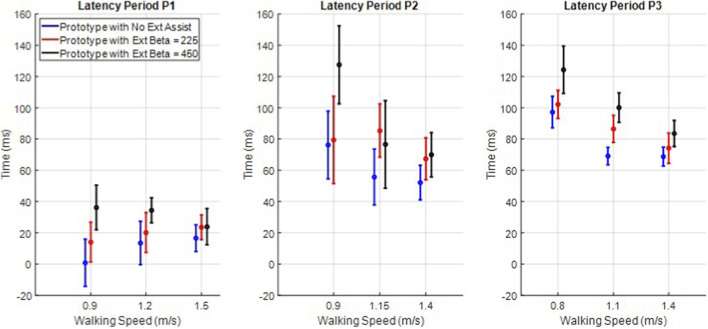
Mean and single standard deviation bars of the latency period for all nine experiments for each participant.

The average latency period was computed for each trial, specifically as the time between when the knee angle crossed 5 deg knee flexion and the time when the ground reaction force reached 10% body weight, each utilizing the prototypes onboard sensors for higher accuracy. The mean computed latency period and associated standard deviation is shown for each participant at each speed and with each level of extension assistance in Figure 8. The maximum swing extension assistance increased the latency period in all participants at all speeds relative to walking without swing assistance (all nine differences were significant, based on p < 0.01 in unpaired t-tests with assumed unequal variances). The average increase in the latency period across participants from the no assist condition to the maximum assist condition was 38 ms, 24 ms, and 13 ms at the self-selected, intermediate, and maximum safe speed, respectively. Swing assistance is associated with an increase in the latency period across all participants and all walking speeds.

### 3.7 Swing and step times

**FIGURE 9:**
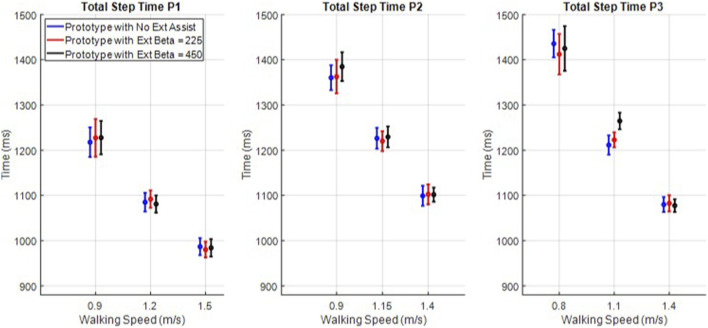
Total step times for all nine trials for each of the three participants.

**FIGURE 10:**
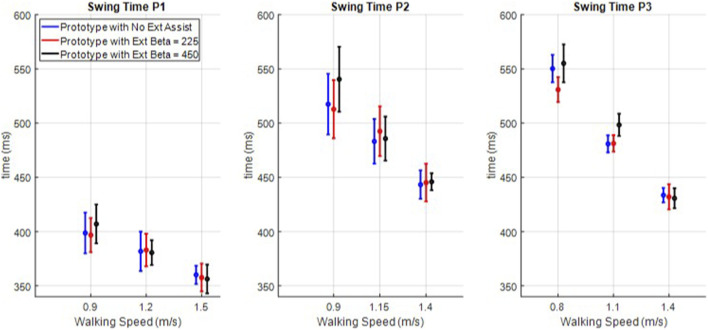
Swing times for all nine trials for each of the three participants.

The knee is shown to extend faster, and the latency period is shown to increase with extension assistance, but this information alone is not enough to establish a causal relationship between the controller and these gait parameters; the user also has control over their swing and step times. To examine the effect of swing extension assistance on swing and step times, the means and single standard deviation bars of step time (Figure 9) and swing time (Figure 10) were computed for each participant and each trial. In the data shown in Figure 9, only two (P3 intermediate speed maximum assistance vs. both other assistance levels) of the 27 cases were statistically different (a significance of 5% with Bonferroni correction of 27), and in Figure 10, only three cases (P3, slow speed half vs. full assistance and intermediate speed full assistance vs. both others) were different. Therefore, although swing extension assistance clearly increased extension velocity and a corresponding increase in latency period, the increased velocity does not appear to have translated to a decrease in swing or step times, particularly at the maximum safe walking speeds.

### 3.8 Participant comments regarding swing extension assistance

A full account of the comments after each trial as well as the participants’ responses to the end of experiment questions is available as Supplementary Material, but illustrative comments about selected walking conditions are included here. When asked about the maximum assistance level at his self-selected walking speed, P2 stated,

“The extension was 100% on. I feel like I did not have to use as much energy to get there [indicating full knee extension] at the same time [as knee loading].”

P3 felt decidedly more negative about the full extension assist at self-selected walking speed, identifying three steps into the trial that extension assistance was on and that he did not like it because of impact at the end of extension (terminal impact). P1 commented that the knee simultaneously felt “heavier” but also that once he got used to it, “it felt like less work”.

At the intermediate walking speed trial with full extension assistance, all three participants identified with certainty that swing extension assistance was on. P1 and P2 both said they liked it, while P3 stated,

“It is definitely on. If I could just take that terminal impact away, I would like it more. But even as is, that was easier walking [than the acclimation trial].”

At the maximum safe walking speed with swing extension assistance, all three participants expressed that they liked the behavior of the knee, relative to the baseline trial, though again P3 indicated that terminal impact was too high. Because P3 completed the no-assistance maximum walking speed trial as his last trial, he was able to retrospectively contradict his prior positive assessment of swing extension assistance with the following statement:

“That was the smoothest trial we have had at this speed… I felt no terminal impact. None. Zero. I really liked this better than those previous [two or three] trials… I do not like the terminal impact [from those prior two or three trials]. It’s exhausting.”

However, P3 reverted to his original assessment of swing extension assistance at the maximum safe walking speed during his summary response to Q1 after all trials were finished. He stated that at the maximum safe walking speed, he felt like the medium assistance level was best, specifically mentioning it would be useful for long distance walking at that speed.

P1 and P2 also noted energetic advantages with swing extension assistance. After all trials were completed, P1 stated,

“[Swing extension assistance at the maximum speed] was very beneficial. I felt less exhausted and more confident.”

P2 echoed a similar sentiment after the end of the trials, explaining that the swing extension assistance was “especially useful for long distances.” After all trials were completed, all three participants noted that the full extension assistance at the self-selected walking pace was too much. P1 described the full extension assistance at self-selected walking pace as, “jarring and unnecessary,” while P2 stated, “the knee was kicking out too hard.” These negative comments about swing extension assistance at their self-selected walking speeds partially contradict their original assessments (immediately following the first three trials). Seemingly, their views of the swing assistance at self-selected walking speeds changed as they became accustomed to walking with and without assistance at faster walking speeds.

## 4 Discussion

This work is the first work to investigate knee prosthesis users walking at their self-identified maximum safe speed and the first work to evaluate swing extension torque during swing extension of fast walking. There are no prior works with which to compare these results. This work demonstrated several noteworthy results on three unilateral prosthesis users, namely, i) all tested users can feel swing extension assistance across speeds including and above their self-selected walking speeds, ii) peak knee extension velocity can be consistently increased by swing extension torque during swing extension, iii) none of the participants utilized this increase in swing extension velocity to quicken their steps; all three instead opted to increase the latency period of their walking, and iv) all three participants complained of an increase in the terminal extension impact, relative to the impact during unassisted trials, indicating that swing extension assistance may require elevated damping at the end of swing extension during fast walking.

### 4.1 Latency period

Swing extension assistance increased the angular velocity of knee extension in all three tested participants. Since both the magnitude and phase of the peak knee flexion angles remained invariant, the increase in knee extension velocity corresponded to reduced time required to achieve full swing extension. A shorter time between the peak knee angle and full knee extension can be used to increase step cadence (i.e., decrease swing time by landing earlier and taking steps more often) or increase the latency period of walking, or a combination of both. All three participants chose to allocate this quicker swing extension to increasing the latency period rather than shortening the swing and stride time.

Why did participants use the increased velocity to increase latency period rather than decrease swing time? The experiments conducted cannot directly address this question, although the authors can conjecture. As (Mâaref et al., 2010) states, the latency period may be indicative of the user’s confidence with the knee; users seem to walk with shorter latency periods when they are more confident in the knee’s behavior. If users were comfortable with their walking conditions, one would expect that the increase in latency period may be unnecessary, and users may opt to take quicker, shorter steps. Alternatively, if users are uncomfortable with the tested walking conditions, they may instead desire the increase in latency period. As the maximum safe walking speed is just that, an uncomfortably fast walking pace for the participants, it is reasonable to expect the participants may want more assurance during swing extension rather than quicker steps.

Additionally, these experiments were conducted on a treadmill with a predetermined walking speed. Participants may have employed a set cadence at a given walking speed, which would have maintained an invariant swing time within that walking speed (assuming other aspects of walking kinematics were also invariant at that speed). The authors did not ask participants to walk faster than the originally determined maximum safe walking speed for their own safety, and because the data from variable walking speeds would be confounded by several other uncontrolled variables and compensatory behaviors. It is impossible to know from these experiments if a longer latency period would have been traded for a shorter swing time if participants were trying to achieve an even faster walking speed. Nonetheless, in the speed constrained case of these experiments, users opted for the increased assurance of latency period rather than adjust their cadence.

### 4.2 Aggregate participant feedback

Overall, all three participants were adept at identifying when the extension assistance was on. P1 successfully identified all nine cases correctly. P2 successfully identified the three cases without extension assistance, and five of the six cases with it (the false negative was a trial with half assistance). P3 successfully identified all three trials of full assistance but was less consistent with the half assistance and no assistance trials, in which he was wrong for one trial and unsure of two more trials.

Because the participants were good at identifying the full swing extension assistance trials from the non-assisted trials, their answers about trial preference may be biased in two ways. Specifically, participants could have been subject to a novelty bias, where they may desire the behavior of the new controller precisely because it is new and different from their daily devices. Alternatively, participants could have been subject to a conservative bias, where they may like the baseline behavior more, precisely because the extension assistance is new and different. Therefore, it is important to evaluate the content of the participants’ statements about why they liked or did not like a trial, rather than only the binary outcome.

All three participants had both positive and negative commentary about the trials with swing extension assistance, relative to the acclimation (non-extension-assisted) control trial. After most extension-assisted trials, P3 commented on the negative sensations of terminal impact. After his final trial (Trial 9, which was his maximum safe walking speed trial without assistance) he even mentioned that the smoothness of the knee without extension assistance outweighed prior positive commentary he had provided about swing extension assistance (though he contradicted this during the four summary questions after completion of the experiment). Although P1 and P2 did not comment on terminal impact during the feedback period after each trial, they each mentioned terminal impact during the four summary questions at the end of the experiment. Specifically, each said that at their self-selected speeds, the knee extension assistance was too much, causing unnecessary terminal impact. As discussed previously, people without amputation who are otherwise healthy do not walk with extension torque during swing extension below about 1.2 m/s. It follows that those with above knee amputations also likely would not benefit from extension assistance at their self-selected walking speeds (0.8–0.9 m/s for these three participants), which aligns with the participant feedback.

At the two faster walking speeds, all three participants mentioned that the swing extension assistance made it easier to walk. P2 and P3 specifically mentioned that they would prefer the extension assistance during extended periods of walking. P2 stated that, while walking with extension assistance, he felt that he was using his prosthesis side hip muscles less. P1 and P2 said that they felt extension assistance allowed them to pick their leg up and it would move for them; they felt that they did not need to use as much motion in their prosthesis-side hip to achieve walking at that speed. Taken together, these comments indicate that all three participants perceived some form of energetic benefit while walking quickly with swing extension assistance, relative to walking without it. Further work is needed to more rigorously qualify and quantify this prospective energetic benefit.

Finally, P1 commented multiple times on what he called his “confidence” in full knee extension. When asked about his feedback for the trial with full extension assistance at his maximum safe walking speed, he said he was “more confident in getting [his] foot out there [and] keeping it there (before heel strike).” It is possible that this “confidence” mentioned by P1 is the result of the biofeedback provided by the increased terminal impact (i.e., P1 may have more strongly felt the knee hit the end of extension and therefore felt a stronger sense that his leg was ready for loading). However, as presented in Figure 8, this trial was also associated with a statistically significant increase to P1’s latency period. Therefore, it is possible this “confidence” was the direct result of the increased latency period, as postulated by (Mâaref et al., 2010). Without more experiments with a modified actuator that reduces or eliminates the elevated sensation of terminal impact, it is impossible to know which behavior caused this increased confidence for P1.

### 4.3 Terminal impact

The addition of powered extension assistance increased the angular momentum of swing extension and as a result the associated terminal impact at the end of swing phase. This terminal impact was a disturbance to the participants in the study, which likely confounded their preference for or against the other biomechanical effects of swing assistance during fast walking. In particular, P3 directly stated “If I could just take that terminal impact away, I would like [the extension assistance] more.”

In hydraulic knees (like the prototype used here), terminal impact is mitigated by a “hydraulic cushion” (sometimes called progressive porting in MPKs) that is designed into the knee hydraulics. The hydraulic cushion employed in the presented knee prototype was not designed with the intention of providing powered swing extension assistance, and was not designed to cushion the increased knee angular velocity (and momentum) associated with it. Before the experiments, it was assumed that any increase in terminal impact caused by extension assistance would be negligible, given the natural speed adaptability of hydraulic systems (resistance to motion increases with velocity). This proved to be false, as the high terminal impact was noted by all three participants, and P3 even stated that he would prefer no assistance at all if terminal impact could not be made more comfortable.

If the knee were redesigned with the additional knee angular momentum considered, the hydraulic cushion could potentially mitigate the additional impact that all three participants disliked. These modifications require a redesign of several components in the prototype’s actuator, and they are planned as future work.

### 4.4 Limitations

There are several limitations of this work which should be understood alongside the results and discussion. First, our experimental procedure intentionally did not randomize the walking speeds alongside the tested swing extension assistance. We chose to complete the trials in ascending walking speed order to provide participants with additional familiarity with the knee’s behavior before asking them to walk at their maximum safe walking speed, for additional safety. Walking speed is non-blindable to the participants (they can immediately tell how fast they are walking), but as indicated by the apparent shift throughout the experiments of the participant responses to their preference for or against swing extension assistance, this choice may have influenced participant feedback on the knee’s behavior. Future work is needed to evaluate participant preferences at high speeds before they become accustomed to the extension controller. Additionally, as previously discussed, the knee’s terminal impact damping was too low, which influenced participant feedback about the extension controller. Also, more participants are needed to make population-wide statistical inferences about the walking controller. This work is only able to comment on the controller’s impact on these three participants. Finally, this work was completed on a treadmill at fixed speeds. Overground walking should be explored, including the possibility that the extension assistance may change participants’ self-selected or maximum safe walking speeds. This was omitted because of safety concerns in these experiments but overground harnesses could be used in future work.

### 4.5 Concluding comments

In this work, the authors explore the effect of layering power-assisted swing extension onto passive knee extension on three participants with transfemoral amputation during fast walking. Powered swing extension assistance increased swing extension velocity and the latency period during fast walking; however, these increases did not translate to faster step times. As such, it appears that users employed the faster swing extension to increase assurance of full knee extension (i.e., latency period) rather than to potentially increase cadence (i.e., reduced swing time).

All three participants expressed a preference for swing extension assistance at their maximum safe walking speeds, relative to the no extension assistance acclimation trial. However, especially at their self-selected walking speed, all three participants mentioned exaggerated terminal impact as a negative side effect of extension assistance during late swing. A redesign of the experimental prototype is underway to explore design improvements that will reduce the sensation of terminal impact. The authors expect that users can receive the prospective benefits of swing extension assistance without a trade-off with terminal impact if the knee is designed to accommodate the additional kinetic energy associated with such assistance, particularly at fast walking speeds.

## Data Availability

The original contributions presented in the study are included in the article/Supplementary Material. Processed but otherwise unparsed and unfiltered time series Visual3D data are provided at the following Digital Object Identifier (DOI): 10.5281/zenodo.18675888. Further inquiries can be directed to the corresponding author.

## References

[B1] Baimyshev A. Lawson B. Goldfarb M. (2018). “Design and preliminary assessment of lightweight swing-assist knee prosthesis,” in 2018 40th annual international conference of the IEEE engineering in medicine and biology society (EMBC). 2018 40th annual international conference of the IEEE engineering in medicine and biology Society (EMBC) (Honolulu, HI: IEEE), 3198–3201. 10.1109/EMBC.2018.8513087 30441073

[B2] Bartlett H. L. King S. T. Goldfarb M. Lawson B. E. (2022). Design and assist-as-needed control of a lightly powered prosthetic knee. IEEE Trans. Med. Robotics Bionics 4 (2), 490–501. 10.1109/TMRB.2022.3161068

[B3] Best T. K. Welker C. G. Rouse E. J. Gregg R. D. (2023). Data-Driven variable impedance control of a powered knee–ankle prosthesis for adaptive speed and incline walking. IEEE Trans. Robotics 39 (3), 2151–2169. 10.1109/TRO.2022.3226887 37304232 PMC10249435

[B4] Boonstra A. Schrama J. Fidler V. Eisma W. H. (1994). The gait of unilateral transfemoral amputees. J. Rehabilitation Med. 26 (4), 217–223. 10.2340/165019771994264217223 7878397

[B5] Camargo J. Ramanathan A. Flanagan W. Young A. (2021). A comprehensive, open-source dataset of lower limb biomechanics in multiple conditions of stairs, ramps, and level-ground ambulation and transitions. J. Biomechanics 119, 110320. 10.1016/j.jbiomech.2021.110320 33677231

[B6] Culver S. C. Vailati L. G. Goldfarb M. (2022). A power-capable knee prosthesis with ballistic swing-phase. IEEE Trans. Med. Robotics Bionics 4 (4), 1034–1045. 10.1109/TMRB.2022.3216475

[B7] Frigo C. Tesio L. (1986). Speed-Dependent variations of lower-limb joint angles during walking. A graphic computerized method showing individual patterns. Am. J. Phys. Med. and Rehabilitation 65 (2), 51–62. 3963165

[B8] Fu G. Zhu J. Wang Z. Mai J. Wang Q. (2023). Mechatronic design and implementation of a low-noise powered knee prosthesis with high backdrivability. IEEE/ASME Trans. Mechatronics 28 (6), 3180–3190. 10.1109/TMECH.2023.3257194

[B9] Gehlhar R. Tucker M. Young A. J. Ames A. D. (2023). A review of current state-of-the-art control methods for lower-limb powered prostheses. Annu. Rev. Control 55, 142–164. 10.1016/j.arcontrol.2023.03.003 37635763 PMC10449377

[B10] Genin J. J. Bastien G. J. Franck B. Detrembleur C. Willems P. A. (2008). Effect of speed on the energy cost of walking in unilateral traumatic lower limb amputees. Eur. J. Appl. Physiology 103 (6), 655–663. 10.1007/s00421-008-0764-0 18478251

[B11] Hagberg K. Brånemark R. (2001). Consequences of non-vascular trans-femoral amputation: a survey of quality of life, prosthetic use and problems. Prosthetics Orthotics International 25 (3), 186–194. 10.1080/03093640108726601 11860092

[B12] Highsmith M. J. Schulz B. W. Hart-Hughes S. Latlief G. A. Phillips S. L. (2010). Differences in the spatiotemporal parameters of transtibial and transfemoral Amputee gait. JPO J. Prosthetics Orthot. 22 (1), 26–30. 10.1097/JPO.0b013e3181cc0e34

[B13] Hood S. A. Lenzi T. (2018). “Preliminary analysis of positive knee energy injection in A transfemoral amputee walking with A powered prosthesis,” in 2018 40th annual international conference of the IEEE engineering in medicine and biology Society (EMBC). 2018 40th annual international conference of the IEEE engineering in medicine and biology Society (EMBC) (Honolulu, HI: IEEE), 1821–1824. 10.1109/EMBC.2018.8512726 30440749

[B14] Hood S. Ishmael M. K. Gunnell A. Foreman K. B. Lenzi T. (2020). A kinematic and kinetic dataset of 18 above-knee amputees walking at various speeds. Sci. Data 7 (1), 150. 10.1038/s41597-020-0494-7 32439980 PMC7242470

[B15] Ingraham K. A. Fey N. P. Simon A. M. Hargrove L. J. (2016). “Assessing the relative contributions of active ankle and knee assistance to the walking mechanics of transfemoral amputees using a powered prosthesis,” Plos One, 11, e0147661. 10.1371/journal.pone.0147661 26807889 PMC4725744

[B16] Latt M. D. Menz H. B. Fung V. S. Lord S. R. (2007). Walking speed, cadence and step length are selected to optimize the stability of head and pelvis accelerations. Exp. Brain Res. 184 (2), 201–209. 10.1007/s00221-007-1094-x 17717650

[B17] Lee J. Goldfarb M. (2023). The effects of swing assistance in a microprocessor-controlled transfemoral prosthesis on walking at varying speeds and grades. Wearable Technol. 4, e9. 10.1017/wtc.2023.4 38487774 PMC10936271

[B18] Lee J. T. Bartlett H. L. Goldfarb M. (2020). Design of a semipowered stance-control swing-assist transfemoral prosthesis. IEEE/ASME Trans. Mechatronics 25 (1), 175–184. 10.1109/TMECH.2019.2952084 33746502 PMC7977329

[B19] Mâaref K. Martinet N. Grumillier C. Ghannouchi S. André J. M. Paysant J. (2010). Kinematics in the terminal swing phase of unilateral transfemoral amputees: Microprocessor-Controlled *versus* swing-phase control prosthetic knees. Archives Phys. Med. Rehabilitation 91 (6), 919–925. 10.1016/j.apmr.2010.01.025 20510984

[B20] Marsh D. Puliti M. Goldfarb M. (2024a). “A novel swing assistance control approach for a powered transfemoral prosthesis,” in IEEE RAS EMBS 10th international conference on biomedical robotics and biomechatronics (Heidelberg, Germany), 134–139.

[B21] Marsh D. Puliti M. Goldfarb M. (2024b). A swing-assist controller for enhancing knee flexion in a semi-powered transfemoral prosthesis. IEEE Trans. Neural Syst. Rehabilitation Eng. 32, 4052–4062. 10.1109/TNSRE.2024.3495517 39527421

[B22] Mochon S. McMahon T. A. (1980a). Ballistic walking. J. Biomechanics 13 (1), 49–57. 10.1016/0021-9290(80)90007-X 7354094

[B23] Mochon S. McMahon T. A. (1980b). Ballistic walking: an improved model. Math. Biosci. 52 (3–4), 241–260. 10.1016/0025-5564(80)90070-X

[B24] Park J. Yoon G. H. Kang J. W. Choi S. B. (2016). Design and control of a prosthetic leg for above-knee amputees operated in semi-active and active modes. Smart Mater. Struct. 25 (8), 085009. 10.1088/0964-1726/25/8/085009

[B25] Sup F. Bohara A. Goldfarb M. (2008). Design and control of a powered transfemoral prosthesis. Int. J. Robotics Res. 27 (2), 263–273. 10.1177/0278364907084588 19898683 PMC2773553

[B26] Toderita D. Favier C. D. Milandri G. S. Vardakastani V. Henson D. P. Bull A. M. (2025). The biomechanical effect of different spring extension assist mechanisms in mechanical 4-bar polycentric prosthetic knees for unilateral above/through-knee amputees. Prosthetics and Orthot. Int. Prepr. 49, 508–514. 10.1097/PXR.0000000000000448 40209768 PMC12509433

[B27] Tran M. Gabert L. Hood S. Lenzi T. (2022). A lightweight robotic leg prosthesis replicating the biomechanics of the knee, ankle, and toe joint. Sci. Robotics 7 (72), eabo3996. 10.1126/scirobotics.abo3996 36417500 PMC9894662

[B28] Tucker M. R. Olivier J. Pagel A. Bleuler H. Bouri M. Lambercy O. (2015). Control strategies for active lower extremity prosthetics and orthotics: a review. J. NeuroEngineering Rehabilitation 12 (1), 1. 10.1186/1743-0003-12-1 25557982 PMC4326520

