# Supplementary material for: Fast walking with swing extension assistance in a nominally passive semi-powered prosthetic knee
Source: Front Robot AI. 2026 Mar 19;13:1747721. doi: 10.3389/frobt.2026.1747721 (PMC13045061; doi:10.3389/frobt.2026.1747721)
Supplement: Supplementary file 1 [file Supplementaryfile1.docx]

Supplementary Material

# Full Participant Comments After Each Trial and During the End of Experiment Summary Questions

The below three tables, Table 1.1, Table 1.2, and Table 1.3, provide participant comments from after each trial as well as their answers to the four “end of experiment” questions described in the paper. The comments after each trial are presented with as few edits as possible, though some were necessary to exclude personally identifying information and unrelated conversation or to add context to enhance understanding of the comments. The end of experiment question answers were not captured during video recording of the experiments, so answers to these answers are transcribed from handwritten records of participants’ answers.

Table 1.1. All Comments During and After the Experiments for Participant 1

| **Participant 1** | | | |
| --- | --- | --- | --- |
| **Trial ID** | **Walking Speed** | **Ext. Gain** | **Comments** |
| 1 | Self | 0 | “I feel pretty confident extension assistance is not on.” |
| 2 | Self | 225 | “Pretty confident that it is on, and I like it a pretty good amount above baseline.” |
| 3 | Self | 450 | “I can 100% tell that it is on. It kinda feels heavier, but when you get used to it, you can …. just let your leg go forward and time it so that you basically just put the knee down. It feels like less work. It feels like we are getting close to a sweet spot. But, the C-Leg has this soft stop [that the prototype leg] does not have.” |
| 4 | Int. | 450 | “I can tell it’s on 100% and I like it.” |
| 5 | Int. | 0 | “I’m pretty sure there is like no extension, it feels kind of like a C-Leg I guess. I feel like I like it the same at the baseline. I think I like some extension assistance, I think it makes me feel more confident in walking.” |
| 6 | Int. | 225 | “Assistance was definitely on. I like it more [than the baseline trial].” |
| 7 | Max | 0 | “I don’t think it is on, and I like this maybe just right above the baseline. Just a hair maybe.” |
| 8 | Max | 450 | “Yes, it’s definitely on. I don’t think it is too much assistance. At that speed, the baseline is just so hard. I definitely like it a lot more than baseline. And then like in my head I am thinking, last trial versus this one… I think I like [this one] better. I feel more confident and it is easier to go at that speed with that amount of extension. I am more confident in getting my foot out there (before heel strike)[and] keeping it there.” |
| 9 | Max | 225 | “I could tell it was on for sure. I feel like it was less aggressive than the last [trial]. At this walking speed, the previous [trial] may have been the sweet spot. You are just. You are going so fast you really want it. If you are going to keep that pace at least. [I like that level of assistance], just not at slow speeds.” |
| **End of Experiment Questions** | | | |
| Q1) Overall thoughts? | | | The participant noted both the flexion and extension assistances were beneficial. |
| Q2) Good prototype behavior? | | | The participant noted that the flexion assistance made the prosthesis feel lighter and smoother during swing phase. He noted that the extension assistance gave him more “confidence” that the foot was ready for weight. At high speed, he stated that extension assistance was “very beneficial. I felt less exhausted and more confident.” |
| Q3) Bad prototype behavior? | | | During the self-selected speed trial, the high extension setting was “jarring and unnecessary. Even for older patients, still may be too much. ” |
| Q4) Any final thoughts? | | | The participant noted that the knee does not “pop out” as fast as the C-Leg 4 during extension without power. |

Table 1.2. All Comments During and After the Experiments for Participant 2

| **Participant 2** | | | |
| --- | --- | --- | --- |
| **Trial ID** | **Walking Speed** | **Ext. Gain** | **Comments** |
| 1 | Self | 225 | “I feel like I don’t have to use as much energy… All I had to do is pick my leg up and it kind of… kind of goes itself. So, I would say especially for like longer distances walking that would actually help out tremendously. It feels different to like powered knees. It’s kind of hard to explain. I feel like anytime I want to stop or go slower or faster I can and with the power knee it feels more like it’s walking for me. It’s got a real robotic feeling. This isn’t as robotic feeling. And you don’t hear [the prototype, like you do powered knees].” |
| 2 | Self | 0 | “I don’t think extension assist was on. Seemed like the first [i.e., the acclimation] trial.” |
| 3 | Self | 450 | “The extension [assist] was 100% on. I feel like I didn’t have to use as much energy to get there at the same time (while gesturing to his outstretched knee, indicating full knee extension). I felt like I can control it better.” |
| 4 | Int. | 225 | “I think extension was on but I’m not sure. It was fine. About the same as the first [acclimation] trial.” |
| 5 | Int. | 450 | “I am sure extension assist was on and I liked it.” |
| 6 | Int. | 0 | “I did not feel any assistance.” |
| 7 | Max | 0 | “I did not feel any assistance. I am indifferent to it, relative to the acclimation trial.” |
| 8 | Max | 450 | “I am sure [extension assistance] was there and I liked it. I am starting to get used to how it feels. I can pick my leg up and it just moves under me. ” |
| 9 | Max | 225 | “I don’t think it was on. I am not really sure, but I am neutral to it relative to the first [acclimation trial]” |
| **End of Experiment Questions** | | | |
| Q1) Overall thoughts? | | | The participant liked it. He could see the benefits “for sure.” |
| Q2) Good prototype behavior? | | | The participant noted that he preferred the extension controller over both the prototype’s passive behavior as well as his daily use knee. He noted it could be “especially useful for long distances.” He believed he did not need to use as much hip flexion (the authors do not know if he intended to reference hip posterior torque or hip anterior torque or both.) |
| Q3) Bad prototype behavior? | | | “The slow speed with maximum extension assist was too much. The knee was kicking out too hard.” |
| Q4) Any final thoughts? | | | “Cool idea, [I] like the direction.” |

Table 1.3. All Comments During and After the Experiments for Participant 3

| **Participant 3** | | | |
| --- | --- | --- | --- |
| **Trial ID** | **Walking Speed** | **Ext. Gain** | **Comments** |
| 1 | Self | 0 | “Extension assistance is definitely on. It is different from the baseline trial. But I don’t mind it one way or the other [relative to the baseline trial]. Just different.” |
| 2 | Self | 450 | Three steps into the trial, he stated that he could completely feel the extension, and it was much too much terminal impact. |
| 3 | Self | 225 | “I felt some extension I think. There was a little bit of bang there (referencing terminal impact) but I don’t think it was much more significant than baseline.” |
| 4 | Int. | 0 | “No different from baseline.” |
| 5 | Int. | 225 | “The terminal impact was still just a little bit higher than I would like. I felt the flexion but the extension I felt that too, but I couldn’t tell if it was because of the flexion was turned on.” |
| 6 | Int. | 450 | “It is definitely on. If I could just take that terminal impact away, I would like it more. But even as is, that was easier walking [than the acclimation trial].” |
| 7 | Max | 225 | “I know extension is on and I liked how it felt. My only reservation is that the extension impact is still a little much.” |
| 8 | Max | 450 | “I couldn’t tell a lot of difference between the last [trial] and this one. Hardly any. There was just a little bit of difference but I can’t put my finger on what it was.” |
| 9 | Max | 0 | “ok I am not positive but I think the extension was turned down or off, and that was the smoothest trial we have had at this speed. Whatever was turned on was perfect for me. If nothing was turned on then I think I like this the most anyways. I felt no terminal impact. None. Zero. I really liked this better than those previous [two or three] trials… I don’t like the terminal impact [from those prior two or three trials]. It’s exhausting” |
| **End of Experiment Questions** | | | |
| Q1) Overall thoughts? | | | Liked extension assist, but terminal impact needs to be lower or he would prefer no assistance at all at higher speeds. For extended walking, he really likes the medium extension assistance setting at 1.4 m/s. |
| Q2) Good prototype behavior? | | | On the fast trials, he feels like he is using less energy with extension controller on. |
| Q3) Bad prototype behavior? | | | “Hard impacts were bad.” |
| Q4) Any final thoughts? | | | “No.” |
